# Characterization of *bla*_NDM_ in two *Escherichia coli* ST1193 clinical isolates in the Gulf region

**DOI:** 10.1093/jacamr/dlae166

**Published:** 2024-11-06

**Authors:** Clement Kin-Ming Tsui, Fatma Ben Abid, Christi Lee McElheny, Manal M Hamed, Andres Perez-Lopez, Ali S Omrani, Yohei Doi

**Affiliations:** Infectious Disease Research Laboratory, National Centre for Infectious Diseases, Singapore 308442, Singapore; Lee Kong Chian School of Medicine, Nanyang Technological University, Singapore 308232, Singapore; Division of Infectious Diseases, Faculty of Medicine, University of British Columbia, Vancouver, Canada V6T 1Z3; Department of Medicine, Weill Cornell Medicine—Qatar, Doha, Qatar; Communicable Diseases Center, Hamad Medical Corporation, Doha, Qatar; Division of Infectious Diseases, Department of Medicine, Hamad Medical Corporation, Doha, Qatar; College of Medicine, Qatar University, Doha, Qatar; Division of Infectious Diseases, University of Pittsburgh School of Medicine, Pittsburgh, USA; Department of Laboratory Medicine and Pathology, Hamad Medical Corporation, Doha, Qatar; Department of Medicine, Weill Cornell Medicine—Qatar, Doha, Qatar; Division of Microbiology, Department of Pathology, Sidra Medicine, Doha, Qatar; Communicable Diseases Center, Hamad Medical Corporation, Doha, Qatar; Division of Infectious Diseases, Department of Medicine, Hamad Medical Corporation, Doha, Qatar; College of Medicine, Qatar University, Doha, Qatar; Division of Infectious Diseases, University of Pittsburgh School of Medicine, Pittsburgh, USA; Departments of Microbiology and Infectious Diseases, Fujita Health University School of Medicine, Toyoake, Japan

## Abstract

**Introduction:**

*Escherichia coli* ST1193 is an emerging high-risk clone associated with the production of plasmid-mediated CTX-type extended-spectrum β-lactamases. However, this clone has seldom been found to contain plasmids carrying carbapenemase genes. We report two epidemiologically unlinked multidrug-resistant (MDR) clinical isolates of *E. coli* ST1193 with plasmids harbouring NDM-type carbapenemase genes from the Gulf region.

**Methods:**

The isolates were identified by MALDI-TOF MS and antibiotic susceptibility testing was performed using the VITEK 2/Phoenix system. A conjugation experiment was performed to assess the transferability of the resistance plasmids. Genomic DNA of both isolates was subject to Illumina sequencing; one isolate was also sequenced using Oxford Nanopore technology. Bioinformatics analyses were performed to detect antimicrobial resistance genes, and to annotate the genetic context of the NDM genes.

**Results and Conclusions:**

Both isolates were resistant to carbapenems using phenotypic tests. Conjugation experiment confirmed that NDM-5-encoding plasmids of both strains could be transferred to the recipient cells. The completed NDM-5-encoding plasmid of *E. coli* isolate FQ71 was highly similar to several plasmids from ST410 isolates in the NCBI database. Genomic analysis revealed the presence of transposase genes and transposons in the flanking regions of the NDM genes in the plasmids. Since carbapenems constitute first-line agents for the treatment of serious infections caused by ESBL producers, *E. coli* ST1193 isolates co-producing ESBL and NDM-type carbapenemases represent a serious challenge for antimicrobial stewardship and infection control programmes.

## Introduction

*Escherichia coli* are a major cause of bloodstream and urinary tract infections. ESBL-producing Enterobacterales such as *E. coli* are found globally in hospital and community settings and represent a major healthcare challenge due to limited effective treatment options.^1,2^ Several *E. coli* lineages such as ST131, ST69, ST410 and ST1193 are considered high-risk clones with an enhanced ability to spread and persist with increased virulence and antimicrobial resistance (AMR).^3–7^ ST1193 is an emerging high-risk clone due to its frequent association with the CTX-M-15 ESBL gene encoding resistance to most penicillins and cephalosporins; ST1193 was the fifth most common ST (behind ST131, ST73, ST69 and ST95) detected among ESBL-producing *E. coli*, and second most common AMR clone (behind ST131) in a geographically well-defined human population.^3^ Although carbapenem resistance in *E. coli* is mainly attributed to co-production of NDM-type and KPC-type carbapenemases by ESBL-producing strains, these genes have seldom been found in ST1193 strains. In the Arabian Peninsula, NDM-1 and KPC-2 genes have been reported in *E. coli* from Oman,^8,9^ but ESBL-producing ST1193 strains harbouring carbapenemase genes have not been identified in Qatar.^10^ Previously, we discovered two MDR *E. coli* ST1193 strains carrying the NDM gene along with the CTX-M-15 gene recovered from two epidemiologically unlinked adult patients during our investigation of carbapenem-resistant (CR) Enterobacterales at a tertiary hospital in Qatar.^11^ This study aimed to elucidate the genetic architecture, mechanisms underlying the spread of the resistance plasmids carrying NDM genes and their transferability in two *E. coli* ST1193 isolates.

## Methods

### Isolation, identification and antimicrobial susceptibility testing

The isolates were obtained as part of a clinical study on carbapenemase-producing Gram-negative bacilli in Qatar.^11^ Both FQ14 and FQ71 were isolated from blood of two unlinked patients and they were identified using the MALDI-TOF MS Biotyper system (Bruker, Bremen, Germany). Antimicrobial susceptibility testing was performed using Vitek 2 (bioMérieux, Marcy-l'Étoile, France) and BD Phoenix (Becton, Dickinson and Company, NJ, USA) automated identification and susceptibility testing systems. Minimum inhibitory concentrations and breakpoints were interpreted according to the Clinical Laboratory Standards Institute 2023.^12^ Isolates were also subjected to susceptibility testing for ertapenem using the standard disc diffusion method.^12^ Isolates with an inhibition zone of ≤21 mm were considered to be suspected carbapenemase producers.^13^

### Conjugation experiment

Conjugation experiments were conducted to assess the transferability of the resistance plasmids.^14^ The experiment was conducted with FQ14 and FQ71 as donors and the sodium-azide-resistant *E. coli* J53^AziR^ as the recipient. First, donor and recipient cells were grown in 10 mL of LB overnight at 37°C. Second, donor cells were mixed with recipients at a ratio of 1:10 in a total volume of 4 mL and incubated at 37°C for 4 h. Third, transconjugants were selected on LB agar containing sodium azide at 100 mg/L and ampicillin at 100 mg/L. Finally, the transfer of a *bla*_NDM_-carrying plasmid was confirmed by positive PCR results for *bla*_NDM_ in the recipient cells. The conjugation frequency was calculated as the ratio of transconjugants to recipient cells.

### Whole genome sequencing and bioinformatic analysis

Genomic DNA were extracted using a DNeasy Blood and Tissue Kit (Qiagen, Hilden, Germany). DNA libraries were constructed with a Nextera DNA library preparation method (Illumina Inc., San Diego, USA), and sequenced on the Illumina NextSeq 550 platform with 2 × 150 cycles (SeqCenter, Pittsburgh, USA). Long-read data for FQ71 were generated using Oxford Nanopore MinION (SQK-LSK109 and flow cell R9.4.1).^15^ MinION reads were generated based on the Guppy software (v.4.0.11) available from Oxford Nanopore Technologies (United Kingdom). The Illumina raw data were assessed by Fastqc (https://www.bioinformatics.babraham.ac.uk/projects/fastqc/), trimmed by Trim Galore v.0.6.3 (http://www.bioinformatics.babraham.ac.uk/projects/trim_galore/), assembled *de novo* using SPAdes v.3.9.0 implemented in shovill (https://github.com/tseemann/shovill).^16^ The assembled genomes were assessed using QUAST v.5.0.2.^17^  *De novo* Illumina-Nanopore assembly for FQ71 was generated with Unicycler v.0.4.7.^18^ ST, plasmids and AMR genes were predicted from the contigs using MLST (https://github.com/tseemann/mlst), the PlasmidFinder v.2.1 and ResFinder v.3.2 databases implemented in ABRicate v.0.9 (https://github.com/tseemann/abricate), based on >70% coverage and 90% sequence identity.^8^ The two assembled genomes were annotated by Bakta,^19^ and the organization of genomes and genes were visualized using Proksee^20^ and Clinker.^21^

## Results and discussion

### Clinical and microbiological characteristics

FQ71 was isolated from the blood of a 16-year-old male with an underlying history of T-cell acute lymphoblastic leukaemia/lymphoma with central nervous system involvement. He was started on the 3B protocol (Cyclophosphamide, Intrathecal MTX and Thioguanine, and two blocks of cytarabine). The chemotherapy cycle was complicated by febrile neutropenia, and he was found to have *E. coli* bacteraemia. He received meropenem, tigecycline and colistin for 3 weeks. The repeated blood cultures after 72 h were negative, and he improved clinically and was discharged home. FQ14 was isolated from the blood of a 76-year-old male with multiple comorbidities including hypertension, diabetes and end-stage renal disease requiring regular haemodialysis via a tunnelled venous catheter. He presented to the emergency department with abdominal pain and vomiting and was found to have *E. coli* in the blood. He received 14 days of extended-infusion meropenem. Follow-up blood cultures were negative, and he was discharged home after clinical improvement. Both isolates were nonsusceptible to meropenem and ertapenem based on an automated susceptibility testing system (data not shown) and disc diffusion test (Table 1).

**Table 1.** Antimicrobial susceptibility tests on different antibiotics using the disc diffusion method

Sample	Date of isolation	Site	ETP	MEM	CIP	CRO	FEP	TZP	AMK	GEN	SXT	FOS	TGC	CL
**FQ14**	13/06/15	Blood	21	22	**6**	**6**	19	**16**	21	21	**6**	34	30	18
**FQ71**	27/10/16	Blood	**16**	20	**6**	**8**	**14**	**12**	18	**6**	**6**	27	23	16

Disc zone size (mm) indicates whether the isolate is resistant (smaller zone size) or sensitive (larger zone size). Values in bold means resistant, and values underlined means intermediate, and the remaining means sensitive. Shaded values had no CLSI guidelines for antibiotic with disc diffusion.

### Genetics and genomic analysis

**Figure 1.**
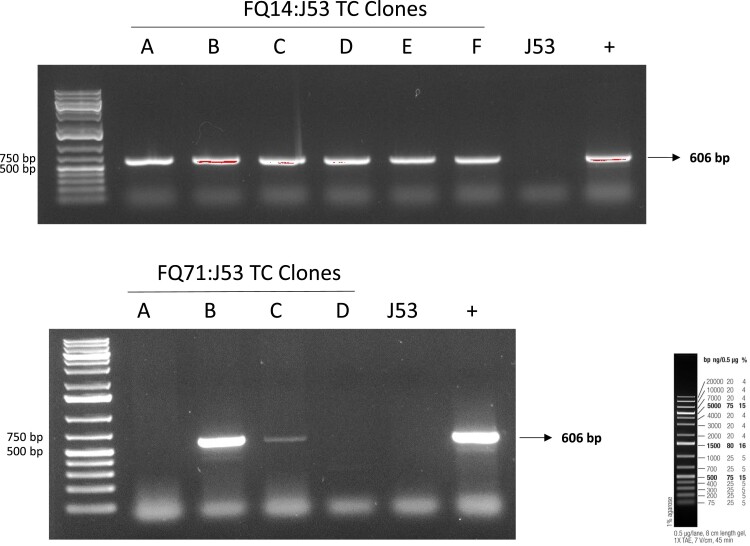
PCR verification of transconjugants (TC) clones of *E. coli* FQ14 and FQ71 with sodium azide-resistant *E. coli* J53^AziR^ as the recipient strain. NDM genes were amplified with primers blaNDM-F-Qatar <ACTTGGCCTTGCTGTCCTT> and blaNDM-R-Qatar <CATTAGCCGCTGCATTGAT>. Positive and negative (J53) controls were included.

In recent years, *bla*_NDM_-producing *E. coli* has been reported across various countries and regions worldwide. We have performed a conjugation experiment and WGS analysis to enhance our understanding of the mechanisms behind the spread of *bla*_NDM_, and to understand the mobile genetic elements that facilitate this transmission and drive the dissemination of resistance across different environments. The conjugation experiment indicated that the *bla*_NDM_-carrying plasmids were able to transfer from both donor strains FQ71 and FQ14 to the recipient *E. coli* J53^AziR^ with ampicillin and sodium azide as selection markers, and the conjugation frequency was 2.8 × 10^−11^ and 1.5 × 10^−8^ per recipient, respectively. PCR also confirmed the presence of *bla*_NDM_ in the recipient cells (Figure 1). Their transferability illustrated a significant risk for the horizontal gene transfer of resistance among ESBL-producing *E. coli* populations. It is possible that our ST1193 isolates acquired a global NDM circulating plasmids from other *E. coli* strains.

**Figure 2.**
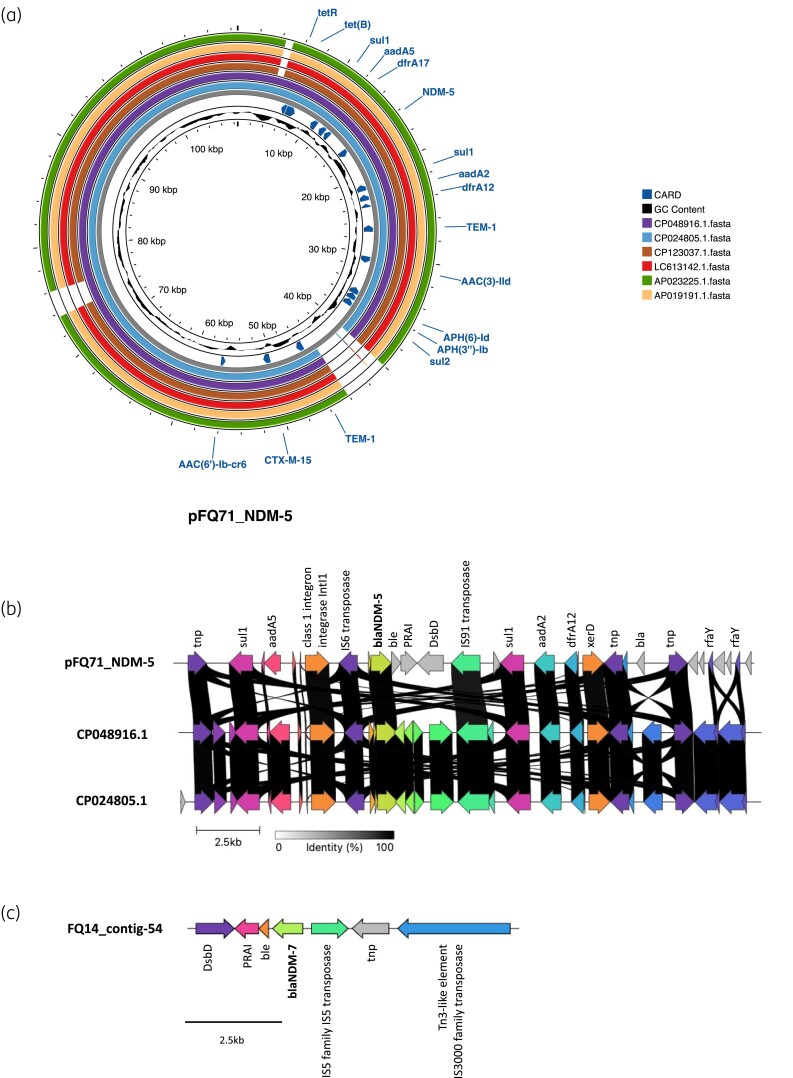
Schematic representation and organization of *bla*_NDM_ on plasmids in FQ71 and FQ14. (a) Genomic comparison of the plasmids bearing *bla*_NDM-5_ in FQ71 and similar plasmids of other ST410 isolates. The circles of different colour represent plasmids in different *E. coli* isolates. (b) Genetic organization of different genes and *bla*_NDM-5_ among FQ71, and two related plasmids (accession no. CP048916.1 and CP024805.1). (c) *bla*_NDM-7_ and flanking genes and transposons on contig 54 in FQ14. The diagram was generated with Proksee and clinker and edited in Microsoft PowerPoint.

*In silico* serotyping of genomes showed that FQ14 and FQ71 were O75:H5 and carried the *fimH*64 allele. Putative virulence-associated genes such as *gad*, *iha*, *iutA*, *ompT*, *sat*, *senB* and *vat* were located on the chromosome. Based on the hybrid genome assembly, FQ71 carried a large multi-replicon plasmid (pFQ71_NDM-5) and three smaller Col-like plasmids ranging from 2.1 to 5.1 kb in size (pFQ71_3, pFQ71_4 and pFQ71_5). *bla*_CTX-M-15_ and *bla*_OXA-1_ were detected on the chromosomal DNA. The largest plasmid sequence in FQ71 harbouring *bla*_NDM-5_ was designated as pFQ71_NDM-5, which is 108 582 bp in size with 4 Inc (FIA, FIB, FII, Q1) replicons detected. BLAST analysis revealed that pFQ71_NDM-5 is similar (99.9%–100% identity and 93%–96% coverage) to several NDM-5 harbouring plasmids in clinical *E. coli* ST410 isolates, such as pE2-NDM-CTX-M (CP048916.1) from Egypt, pAMA1167-NDM-5 (CP024805.1) from Denmark, and pE22P1 (CP123037.1) from China (Figure 2a). The *bla*_NDM-5_ within pFQ71_NDM-5 was flanked by transposases—IS*6* family IS*15DIV* transposase and IS*91* family transposase (Figure 1). Genes encoding bleomycin resistance (*ble*_MBL_), phosphoribosylanthranilate isomerase, Cytochrome c-type biogenesis protein (Dsbd), as well as *sul1*, *aadA2* and *dfr12* were found downstream (Figure 2b). In addition to *bla*_NDM-5_, 16 other resistance genes were detected on pFQ71_NDM5 including *bla*_CTX-M-15_, *bla*_OXA-1_, *bla*_TEM-1B_, *aadA2*, *aadA5*, *aac(3)-IId*, *aac(6′)Ib- D181Y*, *aph(6)-Id*, *aph(3″)-Ib*, *catB3*, *sul1* (two copies), *sul2*, *tet*(B), *dfrA12* and *dfrA17* (Figure 1). The three smaller Col-like plasmids in isolate FQ71 did not carry known resistance genes.

The genome size of FQ14 was ∼5.5 Mb and contained 129 contigs. A BLAST search of contig #54 (8895 bp) containing *bla*_NDM-7_ indicated 100% identity to plasmids of various *Enterobacterales* species (mainly *E. coli*, *n* = 12), which reflects the widespread circulation of such plasmid in the community. Also, *ble*_MBL_, IS*5* transposase and Tn*3*-like transposon genes were located upstream and downstream of *bla*_NDM-7_ (Figure 2c). FQ14 also carried *bla*_CTX-M-15_, *bla*_TEM-1_, *qnrS1*, *aac(6′)-Ib*, *aph(6)-Id*, *aph(3″)-Ib*, *tet(B)*, *sul2* and *dfrA17* on other contigs. Plasmidfinder indicated the presence of Inc types such as IncC, IncFIA, Incl1-1 and IncFIB(AP001918) in FQ14.

Although *E. coli* ST1193 was one of the most common ESBL producers associated with paediatric patients (3.9%) in Qatar, ST1193 was not the most common carbapenemase producer^10^; also only two out 149 CPE producers belonged to *E. coli* ST1193 in adults.^11^ Recent MDR Enterobacterales surveillance study did not detect additional NDM carrying *E. coli* ST1193 in Qatar.^22^ Furthermore, all uropathogenic *E. coli* ST1193 isolates were ESBL producers in Saudi Arabia.^23^ Since NDM-5 is a common variant of NDM in the Middle East region while NDM-7 is not,^9,11,24^ it was possible that these two unlinked ST1193 isolates were introduced sporadically from elsewhere and did not persist and circulate in the local community. Additional WGS investigations for surveillance and infection control would be helpful to determine the persistence and circulation of NDM-producing ST1193. However, our experiments showed that these NDM-encoding plasmids were conjugative and could be disseminated to other bacteria.

In conclusion, our isolates represent the first *E. coli* ST1193 isolates carrying *bla*_NDM_ reported in Qatar. Carbapenemase-producing *Enterobacterales*, especially NDM-producing strains, have become an increasing threat to public health.^1^  *E. coli* typically acquires AMR genes from other members of the *Enterobacterales* via horizontal gene transfer through mobile genetic elements like plasmids.^25,26^ The emergence of CR *E. coli* ST1193 with *bla*_NDM_ located on mobilizable plasmids poses a challenge for antimicrobial stewardship and infection control programmes.

## Date availability

Raw sequence reads are available on NCBI under BioProject accession number PRJNA656934. The genome sequence of FQ71 was submitted to GenBank under accession numbers CP138487-CP138491, JAWVGV000000000.
